# Measuring determinants of implementation behavior: psychometric properties of a questionnaire based on the theoretical domains framework

**DOI:** 10.1186/1748-5908-9-33

**Published:** 2014-03-19

**Authors:** Johanna M Huijg, Winifred A Gebhardt, Elise Dusseldorp, Marieke W Verheijden, Nicolette van der Zouwe, Barend JC Middelkoop, Mathilde R Crone

**Affiliations:** 1 Clinical, Health and Neuropsychology, Leiden University, Wassenaarseweg 52, Leiden, The Netherlands; 2 Netherlands Organization for Applied Scientific Research (TNO), Leiden, Wassenaarseweg 56, Leiden, The Netherlands; 3 Regional Public Health Service Hollands Midden, Parmentierweg 49, Leiden, The Netherlands; 4 Department of Public Health and Primary Care, Leiden University Medical Center, Hippocratespad 21, Leiden, The Netherlands

**Keywords:** Implementation behavior, Determinants, Theoretical Domains Framework, Questionnaire, Physical activity interventions

## Abstract

**Background:**

To be able to design effective strategies to improve healthcare professionals’ implementation behaviors, a valid and reliable questionnaire is needed to assess potential implementation determinants. The present study describes the development of the Determinants of Implementation Behavior Questionnaire (DIBQ) and investigates the reliability and validity of this Theoretical Domains Framework (TDF)-based questionnaire.

**Methods:**

The DIBQ was developed to measure the potential behavioral determinants of the 12-domain version of the TDF (Michie *et al*., 2005). We identified existing questionnaires including items assessing constructs within TDF domains and developed new items where needed. Confirmatory factor analysis was used to examine whether the predefined structure of the TDF-based questionnaire was supported by the data. Cronbach’s alpha was calculated to assess internal consistency reliability of the questionnaire, and domains’ discriminant validity was investigated.

**Results:**

We developed an initial questionnaire containing 100 items assessing 12 domains. Results obtained from confirmatory factor analysis and Cronbach’s alpha resulted in the final questionnaire consisting of 93 items assessing 18 domains, explaining 63.3% of the variance, and internal consistency reliability values ranging from .68 to .93. Domains demonstrated good discriminant validity, although the domains ‘Knowledge’ and ‘Skills’ and the domains ‘Skills’ and ‘Social/professional role and identity’ were highly correlated.

**Conclusions:**

We have developed a valid and reliable questionnaire that can be used to assess potential determinants of healthcare professional implementation behavior following the theoretical domains of the TDF. The DIBQ can be used by researchers and practitioners who are interested in identifying determinants of implementation behaviors in order to be able to develop effective strategies to improve healthcare professionals’ implementation behaviors. Furthermore, the findings provide a novel validation of the TDF and indicate that the domain ‘Environmental context and resources’ might be divided into several environment-related domains.

## Background

Much research and funding is invested into developing, piloting, and evaluating evidence-based innovations to promote health. However, the transfer of effective innovations, such as pharmacological and behavior change interventions, into routine healthcare practice often does not happen as desired [1-5]. With the public health impact of these innovations depending on their implementation in practice, it is important to understand healthcare professionals’ (HCP) implementation behaviors and factors associated with suboptimal use of research evidence [6,7].

Many factors can potentially influence HCPs’ implementation behaviors. These factors may be related to characteristics of the innovation (*e.g*., compatibility, complexity), social setting (*e.g*., norms, support), organizational context (*e.g*., capacity, resources), innovation strategies (*e.g*., training, reimbursement), patient (*e.g*., attitudes, compliance), and the individual HCP (*e.g*., skills, attitudes) [6,8-13]. Identifying the key factors associated with HCP implementation behavior can inform the development of strategies to promote evidence-based behavior [6,14-19].

Research has shown that active implementation strategies, such as educational outreach and reminders, can be effective in enhancing implementation behaviors [20,21]. However, due to the scarce use of theory to inform the choice and design of implementation strategies [22], there is a lack of understanding of why strategies are effective or not [23]. To enhance the effective development of implementation strategies, therefore, many advocate using a theoretical approach to guide the investigation of implementation determinants [14,17,23-25].

Behavior change theories provide testable hypotheses about when and why specific factors will lead to a certain implementation behavior. However, a limitation in the use of these theories to asses and identify factors underlying HCP implementation behavior is the large number of theories that might be used and their overlapping constructs [12,25-27]. The Theoretical Domains Framework (TDF) [28,29] is an integrative framework that can be used to overcome this constraint. Within the original TDF [28], constructs from 33 behavior change theories were grouped into 12 domains of behavioral determinants covering the full range of current scientific explanations for human behavior (*i.e*., ‘Knowledge’, ‘Skills’, ‘Social/professional role and identity’, ‘Beliefs about capabilities’, ‘Beliefs about consequences’, ‘Memory, attention and decision processes’, ‘Environmental context and resources’, ‘Social influences’, ‘Emotion’, ‘Behavioral regulation’, and ‘Nature of the behaviors’). As a consequence, researchers can use this integrative framework instead of having to choose between different theories.

The TDF has instigated a new line of investigation and has been applied in many implementation studies. Specifically, qualitative studies concluded that the TDF was useful for the comprehensive exploration of possible explanations for suboptimal implementation behavior (*e.g*., [30-35]) and for the identification of suitable theories to further investigate these behaviors [27,36]. Furthermore, the framework was used for the development of questionnaires to assess potential implementation behavior determinants [37-39]. So far, however, questionnaires’ internal consistency reliability was insufficient [37-39], and only one out of three questionnaires was able to measure the theoretical domains independently [39]. Consequently, there is need for a valid and reliable method to identify theory-based factors influencing HCPs’ implementation behaviors to be able to design effective implementation strategies [12].

Recently, the TDF [28] has been validated, leading to the revised TDF including 14 domains [29]. Main differences between the original and the revised framework include the separation of the domain ‘Optimism’ from the domain ‘Beliefs about capabilities’ and the domain ‘Reinforcement’ from the domain ‘Beliefs about consequences’. Moreover, the domain ‘Motivation and goals’ was divided into two separate domains, *i.e*., ‘Intentions’ and ‘Goals’, and the domain ‘Nature of the behaviors’ was omitted in the revised framework. As a first step in the development of a TDF-based questionnaire for the valid and reliable assessment of factors influencing HCP implementation behavior, we developed a generic questionnaire assessing the 14 domains of behavioral determinants of the revised TDF [29]. Investigation of questionnaire items’ discriminant content validity based on judgments of a sample of experts on behavior change theory resulted in a questionnaire able to assess all domains discriminately, except for the domains ‘Reinforcement’, ‘Goals’, and ‘Behavioral regulation’. Accordingly, the findings suggested that the 12-domain original version of the TDF [28] might be more applicable in developing a TDF-based questionnaire [40].

The main aim of the current study was to develop a questionnaire based on the 12-domain version of the TDF [28] and to test the psychometric properties of this questionnaire on a sample of HCPs. To validate the Determinants of Implementation Behavior Questionnaire (DIBQ) the following research questions were addressed: 1) does confirmatory factor analysis support the predefined structure of the TDF-based questionnaire (*i.e*., construct validity); 2) is the questionnaire able to measure TDF domains in a reliable way (*i.e*., internal consistency reliability); and 3) are the domains of the questionnaire independently measurable (*i.e*., discriminant validity)? Our specific interest is in HCPs’ implementation of physical activity (PA) interventions, which we used in this study as a field of application for the DIBQ.

## Methods

### Development of the determinants of implementation behavior questionnaire

We developed a questionnaire that initially included 100 items assessing each of the domains through their related key constructs (see Additional file 1). First, constructs within domains were selected based on:

1. Their conceptual relatedness to the content of the domain (*i.e*., Knowledge, Skills, Professional role, and Memory);

2. Their inclusion in relevant theories frequently used in the field of behavior change (and thus ready access to existing items): the Theory of Planned Behavior [41] (*i.e*., Perceived behavioral control, Attitude, Subjective norm, and Intention) and Social Cognitive Theory [42] (*i.e*., Self-efficacy, Outcome expectancies, and Social support);

3. The existence of validated scales to measure constructs (*i.e*., Role clarity, Optimism, Emotions, Action planning, Coping planning, Automaticity); and/or

4. Constructs’ relevance to the implementation of PA intervention in routine healthcare by mapping factors resulting from previous research [13,43] onto the TDF domains (*i.e*., Reinforcement, Priority, Characteristics of the innovation, Characteristics of the socio-political context, Characteristics of the organization, Characteristics of the participants, Characteristics of the innovation strategy, Descriptive norm).

Second, for each domain a minimum of two and a maximum of 24 items were developed, with an average of 4 items for each construct. Items were related to the target behavior ‘delivering PA interventions following the guidelines’. Items measuring the constructs within the domains ‘Knowledge’, ‘Beliefs about capabilities’, ‘Social influences’, ‘Emotion’, ‘Behavioral regulation’, and ‘Nature of the behaviors’ [37,41,42,44-49] were adapted from previously published questionnaires. The content of these items was based on previous research on factors influencing the implementation of PA intervention in routine healthcare [13,43]. For instance, items measuring the constructs Self-efficacy [41] and Coping planning [47] were developed so that they included HCPs’ barriers of lack of time and patient motivation. Items measuring constructs within the domains ‘Skills’, ‘Social/professional role and identity’, ‘Memory, attention, and decision processes’ were based on results of the discriminant content validity study [40]. With regard to the domain ‘Beliefs about consequences’, items measuring the constructs Attitude [41] and Outcome expectancies [42] were adapted from previously published questionnaires, whereas items measuring the construct Reinforcement were newly developed (as none could be located in the literature). Regarding the domain ‘Motivation and goals’, items measuring the construct Intention were adapted from a previously published questionnaire [41], while items were newly developed for the construct Priority. Furthermore, new items were created for the domain ‘Environmental context and resources’. New items were developed based on discussions between WAG, MRC, and JMH. These discussions were informed by the academic literature on the concept and definition of specific domains and constructs, questions to identify behavior change processes as formulated by Michie *et al*. [28], and themes emerging from interviews on the implementation of PA interventions [43]. Finally, the questionnaire was piloted among five colleague researchers and a sample of eight physical therapists. Piloting indicated that the questionnaire was easily understood and well received by the respondents.

### Respondents and procedure

We recruited physical therapists delivering PA interventions to a variety of target groups (*i.e*., people with chronic obstructive pulmonary disease, diabetes, arthritis or obesity). They were recruited through physical therapist associations and contacted opportunistically via their practice websites. Physical therapists were sent an email including the link to the online questionnaire and were assured that their responses would be confidential and anonymous. They reported on their gender, age, practice experience, sort of practice/workplace, and the socioeconomic status (SES) of the majority of their intervention participants. Full questionnaire completion was rewarded with a 25 euro voucher. Non-respondents were sent an email with a questionnaire on their demographic characteristics.

### Data management

Questionnaires were exported from Qualtrics software, version 45433 [50] to IBM SPSS Statistics version 19.0 [51] for analyses. Responses were scored from 1 (strongly disagree) to 7 (strongly agree). Items worded negatively, such as ‘Delivering [PA intervention] following the guidelines is something I often forget’, were reverse-coded. For the six social support items, it was possible to fill in ‘Not applicable’, because not all physical therapists work together with others in delivering PA interventions, and some are part of the management of their organization and therefore do not receive management support. Scores on this category were recoded as missing.

### Data analyses

#### Confirmatory factor analyses

Confirmatory factor analysis was used to examine whether the a priori assignment of items to Michie *et al*.’s [28] TDF domains was supported by the data (*i.e*., research question 1). To perform the confirmatory factor analysis, we used the oblique multiple group (OMG) method [52,53], which has been previously shown to perform better or to be highly comparable to the more well-known confirmatory common factor analysis [54-56]. The OMG method involves calculating correlations between items and domains, from which the following conclusions are drawn: if an item correlates highest with the domain the item was assigned to, the item is correctly assigned to the domain (and the predefined structure is confirmed); if an item correlates highest with a domain the item was not assigned to, the item is incorrectly assigned to the domain (and the predefined structure is not confirmed). In the OMG method, correlations between items and domains are corrected for self-correlation and test length [52].

When an item is assigned incorrectly, adjustments should be made. We used the iterative OMG procedure to make adjustments to the structure of our questionnaire. This step-wise procedure involves testing the adjusted assignment obtained from an OMG analysis in a subsequent OMG analysis on the same data set, which will either support the assignment or provide suggestions for new adjustments. When, based on these suggestions, a new adjustment is made, this assignment can be tested again on the same data set. The iterative procedure continues until the adjusted assignment is supported by the data (*i.e*., items correlate highest with the domain they are assigned to; the adjustment leads to a higher total explained variance) or when none of the adjusted assignments are supported by the data and a newly obtained adjusted assignment is equal to one of the previously assignments. Preferably, changes in item assignment can be justified by a theoretical or conceptual link between the incorrect assigned item and the domain to which it has been assigned [54].

In this study, the iterative procedure of adjustment consisted of two iterations. In the first iteration, adjustments were made based on suggestions from the OMG analyses and theoretical or conceptual links between items and domains. In the second iteration, adjustments were also based on suggestions from the OMG analyses and theoretical or conceptual assumptions. In addition, we compared poor fitting domains from the OMG solution to the solution based on exploratory factor analysis (*i.e*., principal component analysis; PCA [57]) to guide adjustments of the assignment of items to domains. Following the iterative OMG procedure, adjustments were only retained when they were supported by the new results from the OMG analysis. Finally, the variance-accounted-for by the adjusted predefined components was compared to the variance-accounted-for by the components resulting from the PCA. Preferably this difference is small, which indicates that the adjusted predefined structure fits the data well.

#### Internal consistency reliability and discriminant validity

Cronbach’s alpha [58] was computed to assess the internal consistency reliability of the items assessing each domain (*i.e*., research question 2). Two tests of discriminant validity [59] were undertaken to assess if the DIBQ was able to measure the TDF domains discriminately (*i.e*., research question 3). First, discriminant validity was assessed by determining whether the bootstrapped 95% confidence interval around Pearson’s correlations between domains included 1.00 [60]. Second, we calculated attenuation-corrected correlations to discover the ‘true correlation’ between the domains [61].

#### Computational note

The analyses were performed using IBM SPSS statistics version 19.0 [51]. For the OMG analyses, we used a SPSS-macro file obtained from Timmerman and Stuive [62]. Attenuation-corrected correlations were calculated using the R software environment [63] using the R-package Psy [64].

### Ethics

The Medical Ethics Committee of the Leiden University Medical Centre granted ethical approval of this study (reference number NV/CME 09/081).

## Results

### Characteristics of the respondents

Of the 496 physical therapists who were invited for the study, 274 (55.2%) delivering 15 different PA interventions completed the questionnaire. The number of questionnaires analyzed was 270, following removal of physical therapists reporting no experience with PA intervention delivery. Table 1 shows characteristics of respondents and non-respondents. Of the respondents, 58.1% (*n* = 157) were female, they were on average 39.7 (*SD* = 12.3) years old, and had on average 14.9 (*SD* = 11.3) years of practice experience. Most of them worked in a group practice (68.5%, *n* = 185), and most delivered PA interventions to an equal percentage of participants with a low and high SES (53%, *n* = 143) or to people with a low SES (44.8%, *n* = 121). A total of 68 out of 222 non-respondents (30.6%) filled in the non-respondents questionnaire. Comparisons between respondents and non-respondents indicated that the latter were significantly older and had more practice experience.

**Table 1:** Demographic characteristics of respondents and non-respondents

**Demographic variable**	**Respondents (*N*** **= 270)**				**Non-respondents (*N*** **= 68)**			
	**Mean**	**(*SD*)**	***n***	**(%)**	**Mean**	**(*SD *)**	***n***	**(%)**
**Gender**								
Male			113	(12.3)			27	(38.6)
Female			157	(58.1)			39	(55.7)
**Age**	39.7	(12.3)*			45.6	(11.7)*†		
**Practice experience (years)**	14.9	(11.3)*			19.8	(11.8)*‡		
**Sort of practice/workplace**								
Solo practice			7	(2.6)			3	(4.3)†
Duo practice			9	(3.3)			1	(1.4)†
Group practice			185	(68.5)			36	(51.4)†
Multidisciplinary HC center			61	(22.6)			11	(15.7)†
Other			8	(3.0)			4	(5.7)†
**SES intervention participants**								
Mostly high SES			6	(2.2)			4	(5.7)†
50-50			143	(53.0)			30	(42.9)†
Mostly low SES			121	(44.8)			21	(30.0)†

*Note.* Results of chi-square tests and independent t-tests are reported; *,p <0.05; †, based on N = 55; ‡, based on N = 54; HC: Healthcare; SES: Socioeconomic status.

### Psychometric properties of the questionnaire

#### Confirmatory factor analysis

OMG analyses showed that the total variance explained by the initial questionnaire was 48.0%. In other words, the initial assignment of the items to the 12 domains of the TDF explained about half of the total variance in item scores. In the first iteration of adjustments, results of the OMG analysis indicated that model fit could be improved by adjusting the domains ‘Environmental context and resources’ and ‘Beliefs about capabilities’. Based on Fleuren *et al*.’s [8] categorization of innovation determinants into factors related to the innovation, socio-political context, organization, innovation strategy, and Chaudoir *et al*.’s [12] additional category of factors related to the patient, the first adjustment of the questionnaire included dividing the domain ‘Environmental context and resources’ into the domains ‘Innovation’, ‘Socio-political context’, ‘Organization’, ‘Patient’, and ‘Innovation strategy’. This process was done in five subsequent steps (in each step, one new domain was entered), with every step leading to a higher total explained variance, validating the adjustment. With regard to the domain ‘Beliefs about capabilities’, the constructs Self efficacy and Perceived behavioral control did not fit well with the conceptually different ‘Optimism’ items, and therefore ‘Optimism’ items were assigned to a standalone domain. Subsequently, this adjustment was supported by the results of the re-run of the OMG analysis.

In the second iteration, further improvement of model fit was informed by comparing the poor fitting domains from the OMG solution with the solution from the PCA. This led to the assignment of items measuring social support from the management to the domain ‘Organization’, and ‘Priority’ items to a separate domain. Furthermore, the domain ‘Emotion’ was divided into two domains (*i.e*., ‘Negative emotions’ and ‘Positive emotions’) and items measuring the domain ‘Memory, attention, and decision processes’ and the construct Automaticity were combined in the ‘Nature of the behaviors’ domain. Again, these adjustments were validated by re-running the OMG analyses.

For each of the resulting 18 domains, a Cronbach’s alpha was computed. Investigation of ‘alpha, if item deleted’ values revealed that seven items could be deleted. These were one item measuring the domain ‘Priority’, one item measuring the domain ‘Innovation’, three items measuring the domain ‘Organization’, one item measuring the domain ‘Socio-political context’, and one item measuring the domain ‘Patient’. After these adjustments, the final questionnaire included 93 items assessing 18 domains (see Table 2). Definitions of these domains are shown in Table 3. In addition, OMG results showed that the total variance explained by the domains was increased with more than 15% to 63.3%. The variance-accounted-for by the structure of the questionnaire as we built it differed 4.7% with the variance-accounted-for by the components resulting from the PCA. This can be considered a small difference [65], indicating that the predefined (and adjusted) structure fits the data well. A comparison between the initial and the final questionnaire is shown in Table 4.

**Table 2:** Final questionnaire

**Domains**		**Constructs**	**Items**	**Source**
D1	Knowledge	Knowledge (1)	I know how to deliver [PA intervention] following the guidelines.	Adapted from Amemori *et al*. [37]
		Role clarity (3)	Objectives of [PA intervention] and my role in this are clearly defined for me.	Adapted from Wännström [66]
			With regard to [PA intervention], I know what my responsibilities are.	
			In my work with [PA intervention], I know exactly what is expected from me.	
D2	Skills	Skills (3)	I have been trained in delivering [PA intervention] following the guidelines.	New items
			I have the skills to deliver [PA intervention] following the guidelines.	
			I am practiced to deliver [PA intervention] following the guidelines.	
D3	Social/professional role and identity	Professional role (3)	Delivering [PA intervention] following the guidelines is part of my work as a PT.	New items
			As a PT, it is my job to deliver [PA intervention] following the guidelines.	
			It is my responsibility as a PT to deliver [PA intervention] following the guidelines.	
D4	Beliefs about capabilities	Self-efficacy (4)	I am confident that I can deliver [PA intervention] following the guidelines.	Adapted from Content based on Huijg, van der Zouwe *et al*. [43] and Huijg, Gebhardt *et al*. [13]
			I am confident that I can deliver [PA intervention] following the guidelines even when other professionals with whom I deliver [PA intervention] do not do this.	
			I am confident that I can deliver [PA intervention] following the guidelines even when there is little time.	
			I am confident that I can deliver [PA intervention] following the guidelines even when participants are not motivated.	
		Perceived behavioral control (7)	I have control over delivering [PA intervention] following the guidelines.	Adapted from Ajzen [41]
			For me, delivering [PA intervention] following the guidelines is (very difficult – very easy).	
			For me, performing the intake is (very difficult – very easy).	
			For me, delivering the training program is (very difficult – very easy).	
			For me, performing the evaluation is (very difficult – very easy).	
			For me, giving attention to participant’s maintenance of PA behavior outside [PA intervention] is (very difficult – very easy).	
			For me, reporting about the [PA intervention] to the referring professional is (very difficult – very easy).	
D5	Optimism	Optimism (3)	In my work as a PT, in uncertain times, I usually expect the best.	Adapted from Scheier *et al*. [48]
			In my work as a PT, I’m always optimistic about the future.	
			In my work as a PT, overall, I expect more good things to happen than bad.	
D6	Beliefs about consequences	Attitude (4)	For me, delivering [PA intervention] following the guidelines is (not useful at all – very useful).	Adapted from Ajzen [41]
			For me, delivering [PA intervention] following the guidelines is (not worthwhile at all – very worthwhile).	
			For me, delivering [PA intervention] following the guidelines is (not pleasurable at all – very pleasurable).	
			For me, delivering [PA intervention] following the guidelines is (not interesting at all – very interesting).	
		Outcome expectancies (5)	If I deliver [PA intervention] following the guidelines, [PA intervention] will be most effective.	Adapted from Bandura [42]
			If I deliver [PA intervention] following the guidelines, participants will appreciate this.	
			If I deliver [PA intervention] following the guidelines, this will strengthen the collaboration with professionals with whom I deliver [PA intervention].	Content based on Huijg, van der Zouwe *et al*. [43] and Huijg, Gebhardt *et al*. [13]
			If I deliver [PA intervention] following the Guidelines, I will feel satisfied.	
			If I deliver [PA intervention] following the Guidelines, it will help participants to be more physically active.	
		Reinforcement (3)	When I deliver [PA intervention] following the guidelines, I get financial reimbursement.	New items
			When I deliver [PA intervention] following the guidelines, I get recognition from the work context.	Content based on Huijg, van der Zouwe *et al*. [43] and Huijg, Gebhardt *et al*. [13]
			When I deliver [PA intervention] following the guidelines, I get recognition from participants.	
D7	Intentions	Intention (3)	I intend to deliver [PA intervention] following the guidelines in the next three months.	Adapted from Ajzen [41]
			I will definitely deliver [PA intervention] following the guidelines in the next three months.	
			How strong is your intention to deliver [PA intervention] following the guidelines in the next three months?	
D8	Goals	Priority (2)	How often is working on something else on your agenda a higher priority than delivering [PA intervention] following the guidelines?	New items
			How often is working on something else on your agenda more urgent than delivering [PA intervention] following the guidelines?	
D9	Innovation	Innovation characteristics (5)	It is possible to tailor [PA intervention] to participants’ needs?	New items
			It is possible to tailor [PA intervention] to professionals’ needs?	Content based on Rogers [11]
			[PA intervention] costs little time to deliver.	
			[PA intervention] is compatible with daily practice.	
			[PA intervention] is simple to deliver.	
D10	Socio-political context	Socio-political context (3)	Government and local authorities provide sufficient support to interventions such as [PA intervention].	New items
			Insurance companies provide sufficient support to interventions such as [PA intervention].	Content based on Huijg, van der Zouwe *et al*. [43] and Huijg, Gebhardt *et al*. [13]
			PHC is sufficiently oriented towards prevention.	
D11	Organization	Organizational resources and support (4)	In the organization I work, all necessary resources are available to deliver [PA intervention].	New items
			I can count on support from the management of the organization I work in, when things get tough guidelines.	Content based on Huijg, van der Zouwe *et al*. [43] and Huijg, Gebhardt *et al*. [13]
			The management of the organization I work in is willing to listen to my problems with delivering [PA intervention] following the guidelines.	
			The management of the organization I work in is helpful with delivering [PA intervention] following the guidelines.	
D12	Patient	Patient characteristics (2)	Participants of [PA intervention] are motivated.	New items
			Participants of [PA intervention] are positive about [PA intervention].	Content based on Huijg, van der Zouwe *et al*. [43] and Huijg, Gebhardt *et al*. [13]
D13	Innovation strategy	Innovation strategies (7)	[Implementing organization] provides professionals with a training to deliver [PA intervention].	New items
			[Implementing organization] provides the possibility to experience delivering [PA intervention] before professionals need to commit to it.	Content based on Huijg, van der Zouwe *et al*. [43] and Huijg, Gebhardt *et al*. [13]
			[Implementing organization] provides sufficient intervention materials.	
			[Implementing organization] provides assistance to professionals with delivering [PA intervention].	
			[Implementing organization] organizes intervision meetings for professionals.	
			[Implementing organization] provides sufficient financial reimbursement to professionals for [PA intervention] delivery.	
			[Implementing organization] provides insights into results of [PA intervention].	
D14	Social influences	Subjective norm (2)	Most people who are important to me think that I should deliver [PA intervention] following the guidelines.	Adapted from Ajzen [41]
			Professionals with whom I deliver [PA intervention] think I should deliver [PA] intervention] following the guidelines.	
		Descriptive norm (2)	Professionals with whom I deliver [PA intervention] deliver [PA] intervention following the guidelines.	Adapted from Cialdini *et al*. [49]
			Other professionals who work with [PA intervention] deliver [PA intervention] following the guidelines.	
		Social support (3)	I can count on support from professionals with whom I deliver [PA intervention] when things get tough around delivering [PA intervention] following the guidelines.	Adapted from Frese [45]
			Professionals with whom I deliver [PA intervention] are willing to listen to my problems with delivering [PA intervention] following the guidelines.	
			Professionals with whom I deliver [PA intervention] are helpful with delivering [PA intervention] following the guidelines.	
D15	Positive emotions	Positive emotions (6)	When I work with [PA intervention] I feel optimistic.	Adapted from van Veldhoven *et al*. [44]
			When I work with [PA intervention] I feel comfortable.	
			When I work with [PA intervention] I feel calm.	
			When I work with [PA intervention] I feel relaxed.	
			When I work with [PA intervention] I feel cheerful.	
			When I work with [PA intervention] I feel elated.	
D16	Negative emotions	Negative emotions (6)	When I work with [PA intervention] I feel nervous.	Adapted from van Veldhoven *et al*. [44]
			When I work with [PA intervention] I feel pessimistic.	
			When I work with [PA intervention] I feel depressed.	
			When I work with [PA intervention] I feel agitated.	
			When I work with [PA intervention] I feel sad.	
			When I work with [PA intervention] I feel uncomfortable.	
D17	Behavioral regulation	Action planning (3)	I have a clear plan of how I will deliver [PA intervention] following the guidelines.	Adapted from Sniehotta *et al*. [47]
			I have a clear plan under what circumstances I will deliver [PA intervention] following the guidelines.	
			I have a clear plan when I will deliver [PA intervention] following the guidelines.	
		Coping planning (3)	I have a clear plan with regard to delivering [PA intervention] following the guidelines when participants are not motivated.	Adapted from Sniehotta *et al*. [47]
			I have a clear plan with regard to delivering [PA intervention] following the guidelines when there is little time.	Content based on Huijg, van der Zouwe *et al*. [43] and Huijg, Gebhardt *et al*. [13]
			I have a clear plan with regard to delivering [PA intervention] following the guidelines when other professionals with whom I deliver [PA intervention] do not do this.	
D18	Nature of the behaviors	Automaticity (4)	Delivering [PA intervention] following the guidelines is something I do automatically.	Gardner *et al*. [46]
			Delivering [PA intervention] following the guidelines is something I do without having to consciously remember.	
			Delivering [PA intervention] following the guidelines is something I do without thinking.	
			Delivering [PA intervention] following the guidelines is something I start doing before I realize I am doing it.	
		Memory (2)	Delivering [PA intervention] following the guidelines is something I seldom forget.	New items
			Delivering [PA intervention] following the guidelines is something I often forget.	

*Note.* PA: Physical activity; PT: Physical therapist; PHC: Primary healthcare.

**Table 3:** Domain definitions

**Domain**		**Definition**
D1	Knowledge	An awareness of the existence of something.
D2	Skills	An ability or proficiency acquired through practice.
D3	Social/professional and role and identity	A coherent set of behaviors and displayed personal qualities of an individual in a social or work setting.
D4	Beliefs about capabilities	Acceptance of the truth, reality, or validity about an ability, talent, or facility that a person can put to constructive use.
D5	Optimism	The confidence that things will happen for the best or that desired goals will be attained.
D6	Beliefs about consequences	Acceptance of the truth, reality, or validity about outcomes of a behavior in a given situation.
D7	Intentions	A conscious decision to perform a behavior or a resolve to act in a certain way.
D8	Goals	Mental representations of outcomes or end states that an individual wants to achieve.
D9	Innovation	Any characteristics of the innovation that discourages or encourages the development of skills and abilities, independence, social competence, and adaptive behavior.
D10	Socio-political context	Any characteristics of the socio-political context that discourages or encourages the development of skills and abilities, independence, social competence, and adaptive behavior.
D11	Organization	Any characteristics of the organization that discourages or encourages the development of skills and abilities, independence, social competence, and adaptive behavior.
D12	Patient	Any characteristics of the patient that discourages or encourages the development of skills and abilities, independence, social competence, and adaptive behavior.
D13	Innovation strategy	Any characteristics of the innovation strategy that discourages or encourages the development of skills and abilities, independence, social competence, and adaptive behavior.
D14	Social influences	Those interpersonal processes that can cause individuals to change their thoughts, feelings, or behaviors.
D15	Positive emotions	A complex positive reaction pattern, involving experiential, behavioral, and physiological elements, by which the individual attempts to deal with a personally significant matter or event.
D16	Negative emotions	A complex negative reaction pattern, involving experiential, behavioral, and physiological elements, by which the individual attempts to deal with a personally significant matter or event.
D17	Behavioral regulation	Anything aimed at managing or changing objectively observed or measured actions.
D18	Nature of the behaviors	The nature of the aggregate of all responses made by an individual in any situation.

*Note.* All domain definitions, except for the definition of the domain ‘Nature of the behaviors,’ were based on definitions from Cane *et al*. [29], who derived their definitions from the American Psychological Associations’ Dictionary of Psychology [67].

**Table 4:** Comparison between initial and final questionnaire

**Domains**		**Item numbers**	**Amount of items**	**Cronbach’s alpha**	**Explained variance OMG method**	**Explained variance PCA**
		**(from initial questionnaire)**				
D1	Knowledge	1 - 4	4	.93	48.0%	56.5%
D2	Skills	5 - 7	3	.86		
D3	Social/ professional role and identity	8 - 10	3	.90		
D4	Beliefs about capabilities	11 - 24	14	.84		
D5	Beliefs about consequences	25 - 36	12	.83		
D6	Motivation and goals	37 - 42	6	.77		
D7	Memory, attention, and decision p.	43 - 44	2	.71		
D8	Environmental context and res.	45 - 68	24	.82		
D9	Social influences	69 - 78	10	.84		
D10	Emotion	79 - 90	12	.88		
D11	Behavioral regulation	91 - 96	6	.79		
D12	Nature of the behaviors	97 - 100	4	.85		
D1	Knowledge	1 - 4	4	.93	63.3%	68.0%
D2	Skills	5 - 7	3	.86		
D3	Social/ professional role and identity	8 - 10	3	.90		
D4	Beliefs about capabilities	11 - 21	11	.84		
D5	Optimism	22 - 24	3	.79		
D6	Beliefs about consequences	25 - 36	12	.83		
D7	Intentions	37 - 39	3	.91		
D8	Goals	40, 41	2	.88		
D9	Innovation	45 - 49	5	.68		
D10	Socio-political context	51 - 53	3	.73		
D11	Organization	57, 76 - 78	4	.85		
D12	Patient	60, 61	2	.74		
D13	Innovation strategy	62 - 68	7	.82		
D14	Social influences	69 - 75	7	.86		
D15	Positive emotions	80, 82, 84, 87, 89, 90	6	.85		
D16	Negative emotions	79, 81, 83, 85, 86, 88	6	.85		
D17	Behavioral regulation	91 - 96	6	.79		
D18	Nature of the behaviors	43, 44, 97 - 100	6	.85		

*Note.* OMG method, oblique multiple group method; PCA, principal component analysis.

#### Internal consistency reliability and discriminant validity

Internal consistency reliability values for the 18 domains of the final questionnaire ranged from .68 for the domain ‘Innovation’ (*i.e*., the only domain with an alpha < .70) to .93 for the domain ‘Knowledge’. None of the bootstrapped 95% confidence intervals around Pearson’s correlations included 1.00, indicating sufficient discriminant validity (for an overview of all correlations between domains, see Additional file 2). In addition, we found high attenuation-corrected correlations between the domains ‘Knowledge’ and ‘Skills’ (*r* = .80) and the domains ‘Skills’ and ‘Social/professional role and identity’ (*r* = .86), which suggests overlap between these domains (see Additional file 3).

## Discussion

We developed and tested a questionnaire assessing factors influencing HCPs’ implementation behaviors that was based on a theoretical framework of behavioral determinants [28]. The DIBQ was one of the first TDF-based questionnaires that was developed in a rigorous manner, and showing very good psychometric properties. That is, it had good construct validity, and the majority of domains showed high internal consistency reliability and discriminant validity. While our focus was on the measurement of factors influencing the implementation of PA interventions in PHC, we suggest that the DIBQ can be applied more broadly, as the questionnaire can easily be adapted to other contexts in which implementation research takes place. Consequently, the DIBQ can solve previously reported problems with the measurement of theory-based factors underlying HCP behavior [12,25-27]. This can contribute to the development of effective implementation strategies and subsequently the impact of evidence-based interventions.

With regard to the questionnaire’s construct validity, our findings supported the majority of the predefined structure of the questionnaire that was based on the 12 domains of the TDF [28]. They correspond with Taylor *et al*. [39,68], who found good discriminant validity of TDF domains in a questionnaire measuring influences on patient safety behaviors [39] and in the Determinants of Physical Activity Questionnaire [68]. These results provide an additional level of validation for the content of the TDF, and they confirm the viability of using the framework for construction of a theory-based questionnaire. Nevertheless, the questionnaire’s construct validity could be enhanced by some adjustments in content of the domains and the structure of the questionnaire to 18 domains.

The main adjustment we made to the structure of the questionnaire was dividing the domain ‘Environmental context and resources’ into five different environment-related domains: ‘Innovation’, ‘Socio-political context’, ‘Organization’, ‘Patient’, and ‘Innovation strategy’. This adjustment is consistent with leading theoretical models on the introduction of innovations in healthcare [6,8-12]. Replication of this domain-structure in future research may suggest including five different environment-related domains in the TDF. Next, ‘Optimism’ items were separated from the domain ‘Beliefs about capabilities’. This separation makes sense because ‘Optimism’ items were measured as a general disposition (*e.g*., ‘In my work as a physical therapist, in uncertain times, I usually expect the best’), whereas ‘Beliefs about capabilities’ items concerned capabilities that are required to achieve a specific outcome (*e.g*., ‘I am confident that I can deliver [PA intervention] following the guidelines’). Furthermore, the adjustment corresponds with the results of the recent validation of the TDF [29]. Items measuring social support from the management were assigned to the domain ‘Organization’ and ‘Priority’ items were separated from ‘Intention’ items. The first adjustment could also be justified by conceptual links between items and domains, and the latter adjustment corresponded with results of the validated TDF [29]. In addition, dividing the domain ‘Emotion’ into the domains ‘Positive emotions’ and ‘Negative emotions’ could be explained by previous research that indicated that positive and negative affect are two relatively independent constructs that can be measured discriminately [69,70]. Based on similarities in their content, items measuring the domain ‘Memory, attention, and decision processes’ and Automaticity items were merged into the domain ‘Nature of the Behaviors’. Moreover, the link between automatic behaviors and memory was highlighted by Wood and Neal [71]. When developing a TDF-based questionnaire, it is possible that adding questions on attention and decision making to the memory items might decrease the overlap between the domains ‘Memory, attention, and decision processes’ and ‘Nature of the Behaviors’. Finally, some items measuring the domains ‘Priority’, ‘Innovation’, ‘Organization’, ‘Socio-political context’, and ‘Patient’ were deleted based on the domains’ Cronbach’s alpha values. An explanation based on the content of these items could not be found; however, lack of internal consistency reliability of the domains ‘Priority’, ‘Innovation’, ‘Organization’, ‘Socio-political context’, and ‘Patient’ might be related to the fact that the items measuring these domains were all newly developed. This suggests that items measuring the domain ‘Environmental context and resources’ can be improved (see Chaudoir *et al*. [12] for an overview of measures assessing these domains related to the environment).

No adjustments were needed for five out of the 12 domains of the initial questionnaire: ‘Knowledge’, ‘Skills’, ‘Social/professional role and identity’, ‘Beliefs about consequences’, and ‘Behavioral regulation’. This might be explained by the use of previously published questionnaires for the development of ‘Knowledge’ and ‘Behavioral regulation’ items, and most of the ‘Beliefs about consequences’ items. Furthermore, items measuring the domains ‘Skills’ and ‘Social/professional role and identity’ were validated by the discriminant content validity study [40]. Noticeably, the ‘Knowledge’ item ‘I know how to…,’ ‘Reinforcement’ items, and items measuring the construct Action Planning performed well, while they could not be validated by the discriminant content validity study [40]. This might be explained by the divergence in the main aims of the two studies; the increased focus on differences between individual items when investigating items’ discriminant content validity, and the emphasis on similarities between groups of items when examining a questionnaire’s construct validity. Indeed, in the present study, items that were not validated in the discriminant content validity study were surrounded by other previously validated items.

Compared to three other studies using a TDF-based questionnaire to identify implementation behavior determinants [37-39], our questionnaire demonstrated high internal consistency reliability for the majority of domains. Explanations for this might be the lower number of items that the previous studies used to measure each domain [37-39] and the development of items for domains instead of constructs within domains [38,39]. Furthermore, it is not clear to what extent Beenstock *et al*. [38] and Taylor *et al*. [39] used items from previously published questionnaires.

Although OMG analyses revealed sufficient discriminant validity on item level, attenuation-corrected correlations revealed overlap between the domains ‘Knowledge’ and ‘Skills’ and ‘Skills’ and ‘Social/professional role and identity’. On the other hand, bootstrapped 95% confidence intervals around correlations suggested that the questionnaire was able to measure TDF domains discriminately. Based on these results and the different content of the domains, we did not merge them into one single domain. However, high correlations between domains might be problematic when analyzing associations between domains and outcome variables taking a multivariate approach.

While our focus was on the measurement of factors influencing HCPs’ implementation of PA interventions, the questionnaire was designed to be easily adaptable so it can be used in studies investigating implementation behaviors performed by other HCPs in other settings. However, depending on the behavior, the implementing HCP, and the context, it may be necessary to include items for specific barriers and facilitators. For example, time, patient motivation, and financial support may play a role in the delivery of PA interventions by physical therapists, while these factors might not relate to other behaviors, HCPs and settings. Moreover, validity and reliability of use of the questionnaire for other behaviors, HCPs and settings needs further investigation.

Some limitations of this study need to be taken into consideration when interpreting the results. First, respondents were physical therapists delivering PA interventions to a variety of target groups. In this study, we did not distinguish between the different PA interventions. Our results suggest sufficient internal validity of the DIBQ. However, a question remains as to whether the structure of the DIBQ holds for every specific PA intervention. In this study, small sample sizes within each PA intervention (sample sizes varied from 4 to 101) hindered the performance of confirmatory factor analysis for each PA intervention separately. A recommendation for future applications of the DIBQ is to replicate the reliability analysis for the target group at hand. Second, the questionnaire assessed TDF domains through their related constructs. However, to develop a questionnaire that is of an acceptable length to fill in, only a selection of constructs could be measured. Although the selection of key-constructs was based on previous research on factors influencing the implementation of PA interventions in primary healthcare [13,43], it could be that some of the domains’ key-constructs are not part of the questionnaire leading to decreased validity of the measurement of domains. For example, the construct Intrinsic motivation [72] was not included to measure the domain ‘Motivation and goals’ and the construct Burnout [73] was not included to measure the domain ‘Emotion’, although we know from previous research that these are important determinants for HCPs’ evidence-based practice [74,75]. Nevertheless, a questionnaire including 93 items might still be too long to fill in. This could also be an explanation for the 55.2% response rate, which was comparable to previous reported response rates of 54% [76] and 57% [77] in surveys among physical therapists, but can be considered low in comparison to Barrett *et al*. [78], who reached a response rate of 88%. A next step in the development process could be to develop a shorter version of the DIBQ and assess its psychometric properties. One strategy to decrease the amount of items would be to select items measuring the domains directly, instead of through their related key construct. Taking into account the criterion for a reliable component (*i.e*., at least three items with a loading above .80 [79]), this could decrease the average of 4 items for each construct to 4 items for each domain. The results of the discriminant content validity study [40] may guide the selection of items in order to obtain a shortened version of the questionnaire. Comparisons between respondents and non-respondents indicated that the latter were significantly older and had more practice experience, which limits the generalizability of our results. Finally, the methods used to validate our questionnaire were limited to factor analyses and the examination of discriminant validity of the domains, and only internal consistency reliability was assessed. Future research should also investigate items’ predictive validity and test-retest reliability of the questionnaire.

## Conclusions

This study describes the development and initial validation of the DIBQ. The questionnaire showed good construct validity (*i.e*., research question 1) and the majority of domains showed high internal consistency reliability (*i.e*., research question 2) and discriminant validity (*i.e*., research question 3). Therefore, the questionnaire is viable to measure potential determinants of implementation behavior in a theory-based and comprehensive way. The identification of factors influencing implementation behaviors provides important information on what factors should be targeted when designing strategies to promote the effective implementation of interventions [6,14-19]. This is highly likely to increase the impact of health behavior change interventions. Future studies on the psychometric properties of the questionnaire are warranted and should go beyond construct validity, internal consistency reliability, and discriminant validity. In addition, more research is needed to understand the strengths and limitations of the questionnaire when it is used for other behaviors among other HCPs and in other settings.

## Consent

In our study, completion of the questionnaire indicated participants’ consent for their participation in the study.

## Abbreviations

HCP: Healthcare professional; TDF: Theoretical domains framework; PA: Physical activity; SES: Socioeconomic status.

## Competing interests

The authors declare that they have no competing interests.

## Authors’ contributions

JMH was involved in the design of the study, recruited the respondents, collected, analyzed and interpreted the data, and wrote the initial and subsequent drafts of the manuscript. ED was involved in data analysis and interpretation, and commented on the manuscript. WAG and MRC were involved in the conception and the design of the study, assisted with interpretation of the data, and critically revised the manuscript. MWV, NZ and BJCM were involved in the conception and the design of the study, assisted with interpretation of the data, and commented on the manuscript. All authors read and approved the final manuscript.

## Supplementary Material

Additional file 1 Initial questionnaire. Click here for file

Additional file 2 Correlations between domains. Click here for file

Additional file 3 Attenuation-corrected correlations. Click here for file
