# Supplementary material for: Measuring determinants of implementation behavior: psychometric properties of a questionnaire based on the theoretical domains framework
Source: Implement Sci. 2014 Mar 19;9:33. doi: 10.1186/1748-5908-9-33 (PMC4000005; doi:10.1186/1748-5908-9-33)
Supplement: Additional file 1 — Initial questionnaire. [file 1748-5908-9-33-S1.pdf]

## Additional file 1 – Initial questionnaire

| Domain | | Constructs | | Items | Source |
|---|---|---|---|---|---|
| D1 | Knowledge | Knowledge (1) | 1. | I know how to deliver [PA intervention] following the guidelines | Adapted from Amemori et al. [37] |
| | | Role clarity(3) | 2. | Objectives of [PA intervention] and my role in this are clearly defined for me | Adapted from Wännström [66] |
| | | | 3. | With regard to [PA intervention] I know what my responsibilities are | |
| | | | 4. | In my work with [PA intervention] I know exactly what is expected from me | |
| D2 | Skills | Skills (3) | 5. | I have been trained in delivering [PA intervention] following the guidelines | New items |
| | | | 6. | I have the skills to deliver [PA intervention] following the guidelines | |
| | | | 7. | I am practiced to deliver [PA intervention] following the guidelines | |
| D3 | Social/ professional role and identity | Professional role (3) | 8. | Delivering [PA intervention] following the guidelines is part of my work as a PT | New items |
| | | | 9. | As a PT it is my job to deliver [PA intervention] following the guidelines | |
| | | | 10. | It is my responsibility as a PT to deliver [PA intervention] following the guidelines | |
| D4 | Beliefs about capabilities | Self-efficacy (4) | 11. | I am confident that I can deliver [PA intervention] following the guidelines | Adapted from Bandura [42] |
| | | | 12. | I am confident that I can deliver [PA intervention] following the guidelines even when other professionals with whom I deliver [PA intervention] do not do this | Content based on Huijg, van der Zouwe et al. [43] and Huijg, Gebhardt et al. [13] |
| | | | 13. | I am confident that I can deliver [PA intervention] following the guidelines even when there is little time | |
| | | | 14. | I am confident that I can deliver [PA intervention] following the guidelines even when participants are not motivated | |
| | | Perceived Behavioral Control (7) | 15. | I have control over delivering [PA intervention] following the guidelines | Adapted from Ajzen [41] |
| | | | 16. | For me, delivering [PA intervention] following the guidelines is (very difficult – very easy) | |
| | | | 17. | For me, performing the intake is (very difficult – very easy) | |
| | | | 18. | For me, delivering the training program is (very difficult – very easy) | |

| | | | | | |
|---|---|---|---|---|---|
| | | | 19. | For me, performing the evaluation is (very difficult – very easy) | |
| | | | 20. | For me, giving attention to participant's maintenance of PA behavior outside [PA intervention] is (very difficult – very easy) | |
| | | | 21. | For me, reporting about the [PA intervention] to the referring professional is (very difficult – very easy) | |
| | | Optimism (3) | 22. | In my work as a PT, in uncertain times, I usually expect the best | Adapted from Scheier et al. [48] |
| | | | 23. | In my work as a PT, I'm always optimistic about the future | |
| | | | 24. | In my work as a PT, overall, I expect more good things to happen than bad | |
| D5 | Beliefs about consequences | Attitude (4) | 25. | For me, delivering [PA intervention] following the guidelines is (not useful at all – very useful) | Adapted from Ajzen [41] |
| | | | 26. | For me, delivering [PA intervention] following the guidelines is (not worthwhile at all – very worthwhile) | |
| | | | 27. | For me, delivering [PA intervention] following the guidelines is (not pleasurable at all – very pleasurable) | |
| | | | 28. | For me, delivering [PA intervention] following the guidelines is (not interesting at all – very interesting) | |
| | | Outcome expectancies (5) | 29. | If I deliver [PA intervention] following the guidelines [PA intervention] will be most effective | Adapted from Bandura [42] |
| | | | 30. | If I deliver [PA intervention] following the guidelines participants will appreciate this | Content based on Huijg, van der Zouwe et al. [43] and Huijg, Gebhardt et al. [13] |
| | | | 31. | If I deliver [PA intervention] following the guidelines this will strengthen the collaboration with professionals with whom I deliver [PA intervention] | |
| | | | 32. | If I deliver [PA intervention] following the guidelines I will feel satisfied | |
| | | | 33. | If I deliver [PA intervention] following the guidelines it will help participants to be more physically active | |
| | | Reinforcement (3) | 34. | When I deliver [PA intervention] following the guidelines, I get financial reimbursement | New items |
| | | | 35. | When I deliver [PA intervention] following the guidelines, I get recognition from the work context | Content based on Huijg, van der Zouwe et al. [43] and Huijg, Gebhardt et al. [13] |
| | | | 36. | When I deliver [PA intervention] following the guidelines, I get recognition from participants | |
| D6 | Motivation and goals | Intention (3) | 37. | I intend to deliver [PA intervention] following the guidelines in the next three months | Adapted from Ajzen [41] |
| | | | 38. | I will definitely deliver [PA intervention] following the guidelines | |

| | | | | | |
|---|---|---|---|---|---|
| | | | | in the next three months | |
| | | | 39. | How strong is your intention to deliver [PA intervention] following the guidelines in the next three months | |
| | | Priority (3) | 40. | How often is working on something else on your agenda a higher priority than delivering [PA intervention] following the guidelines | New items |
| | | | 41. | How often is delivering [PA intervention] following the guidelines more important than working on something else on your agenda | |
| | | | 42. | How often is working on something else on your agenda more urgent than delivering [PA intervention] following the guidelines | |
| D7 | Memory, attention, and decision processes | Memory (2) | 43. | Delivering [PA intervention] following the guidelines is something I seldom forget | New items |
| | | | 44. | Delivering [PA intervention] following the guidelines is something I often forget | |
| D8 | Environmental context and resources | Characteristics of the innovation (6) | 45. | It is possible to tailor [PA intervention] to participants' needs | New items |
| | | | 46. | It is possible to tailor [PA intervention] to professionals' needs | |
| | | | 47. | [PA intervention] costs little time to deliver | Content based on Rogers [11], Huijg, van der Zouwe et al. [43] and Huijg, Gebhardt et al. [13] |
| | | | 48. | [PA intervention] is compatible with daily practice | |
| | | | 49. | [PA intervention] is simple to deliver | |
| | | | 50. | [PA intervention] has advantages compared to standard care | |
| | | Characteristics of the socio-political context (4) | 51. | Government and local authorities provide sufficient support to interventions such as [PA intervention] | |
| | | | 52. | Insurance companies provide sufficient support to interventions such as [PA intervention] | |
| | | | 53. | PHC is sufficiently oriented towards prevention | |
| | | | 54. | There is a good collaboration between professionals who deliver [PA intervention] | |
| | | Characteristics of the organization (4) | 55. | In the organization I work, are sufficient potential participants of [PA intervention] present | |
| | | | 56. | In the organization I work, is enough time to deliver [PA intervention] | |
| | | | 57. | In the organization I work, all necessary resources are available to deliver [PA intervention] | |
| | | | 58. | In the organization I work, there is a good collaboration between professionals who deliver [PA intervention] | |
| | | Characteristics of the [PA intervention] | 59. | I know participants of [PA intervention] personally | |
| | | | 60. | Participants of [PA intervention] are motivated | |

| | | | | | |
|---|---|---|---|---|---|
| | | participants (3) | | | |
| | | | 61. | Participants of [PA intervention] are positive about [PA intervention] | |
| | | Characteristics of the innovation strategy (7) | 62. | [Implementing organization] provides professionals with a training to deliver [PA intervention] | |
| | | | 63. | [Implementing organization] provides the possibility to experience delivering [PA intervention] before professionals need to commit to it | |
| | | | 64. | [Implementing organization] provides sufficient intervention materials | |
| | | | 65. | [Implementing organization] provides assistance to professionals with delivering [PA intervention] | |
| | | | 66. | [Implementing organization] organizes intervision meetings for professionals | |
| | | | 67. | [Implementing organization] provides sufficient financial reimbursement to professionals for [PA intervention] delivery | |
| | | | 68. | [Implementing organization] provides insights into results of [PA intervention] | |
| D9 | Social influences | Subjective norm (2) | 69. | Most people who are important to me think that I should deliver [PA intervention] following the guidelines | Adapted from Ajzen [41] |
| | | | 70. | Professionals with whom I deliver [PA intervention] think I should deliver [PA] intervention] following the guidelines | |
| | | Descriptive norm (2) | 71. | Professionals with whom I deliver [PA intervention] deliver [PA] intervention following the guidelines | Adapted from Cialdini et al. [49] |
| | | | 72. | Other professionals who work with [PA intervention] deliver [PA intervention] following the guidelines | |
| | | Social support (6) | 73. | I can count on support from professionals with whom I deliver [PA intervention] when things get tough around delivering [PA intervention] following the guidelines | Adapted from Frese [45] |
| | | | 74. | Professionals with whom I deliver [PA intervention] are willing to listen to my problems with delivering [PA intervention] following the guidelines | |
| | | | 75. | Professionals with whom I deliver [PA intervention] are helpful with delivering [PA intervention] following the guidelines | |
| | | | 76. | I can count on support from the management of the organization I work in, when things get tough around delivering [PA intervention] following the guidelines | |
| | | | 77. | The management of the organization I work in is willing to listen to my problems with delivering [PA intervention] following the | |

| | | | | |
|---|---|---|---|---|
| | | | guidelines<br>78. The management of the organization I work in is helpful with delivering [PA intervention] following the guidelines | |
| D10 | Emotion | Emotions (12) | 79. When I work with [PA intervention] I feel nervous<br>80. When I work with [PA intervention] I feel optimistic<br>81. When I work with [PA intervention] I feel pessimistic<br>82. When I work with [PA intervention] I feel comfortable<br>83. When I work with [PA intervention] I feel depressed<br>84. When I work with [PA intervention] I feel calm<br>85. When I work with [PA intervention] I feel agitated<br>86. When I work with [PA intervention] I feel sad<br>87. When I work with [PA intervention] I feel relaxed<br>88. When I work with [PA intervention] I feel uncomfortable<br>89. When I work with [PA intervention] I feel cheerful<br>90. When I work with [PA intervention] I feel elated | Adapted from van Veldhoven [44] |
| D11 | Behavioral regulation | Action planning (3) | 91. I have a clear plan of how I will deliver [PA intervention] following the guidelines<br>92. I have a clear plan under what circumstances I will deliver [PA intervention] following the guidelines<br>93. I have a clear plan when I will deliver [PA intervention] following the guidelines | Adapted from Sniehotta et al. [47] |
| | | Coping planning (3) | 94. I have a clear plan with regard to delivering [PA intervention] following the guidelines when participants are not motivated<br>95. I have a clear plan with regard to delivering [PA intervention] following the guidelines when there is little time<br>96. I have a clear plan with regard to delivering [PA intervention] following the guidelines when other professionals with whom I deliver [PA intervention] do not do this | Adapted from Sniehotta et al. [47]<br><br>Content based on Huijg, van der Zouwe et al. [43] and Huijg, Gebhardt et al. [13] |
| D12 | Nature of the behaviors | Automaticity (4) | 97. Delivering [PA intervention] following the guidelines is something I do automatically<br>98. Delivering [PA intervention] following the guidelines is something I do without having to consciously remember<br>99. Delivering [PA intervention] following the guidelines is something I do without thinking<br>100. Delivering [PA intervention] following the guidelines is something I start doing before I realize I am doing it | Gardner et al. [46] |

*Note.* PA, physical activity; PT, physical therapist; PHC, primary health care
