# Supplementary material for: Measuring determinants of implementation behavior: psychometric properties of a questionnaire based on the theoretical domains framework
Source: Implement Sci. 2014 Mar 19;9:33. doi: 10.1186/1748-5908-9-33 (PMC4000005; doi:10.1186/1748-5908-9-33)
Supplement: Additional file 2 — Correlations between domains. [file 1748-5908-9-33-S2.pdf]

Additional file 2 – Correlations between domains

		D1	D2	D3	D4	D5	D6	D7	D8	D9	D10	D11	D12	D13	D14	D15	D16	D17	D18
D1	Knowledge		.71**	.68**	.35**	.09	.38**	.24**	.17**	.20**	.04	.14*	.14*	.06	.16*	.33**	-.30**	.28**	.25**
D2	Skills			.76**	.40**	.12*	.45**	.36**	.18**	.16**	.02	.13*	.21**	.03	.24**	.32**	-.30**	.39**	.38**
D3	Social/professional R&I				.34**	.08	.46**	.41**	.21**	.15*	.08	.10	.21**	.10	.27**	.26**	-.25**	.32**	.34**
D4	Beliefs about capabilities					.30**	.52**	.40**	.26**	.53**	.12	.32**	.29**	.22**	.41**	.47**	-.35**	.64**	.56**
D5	Optimism						.24**	.08	.06	.21**	.08	.23*	.18**	.02	.16**	.30**	-.15*	.21**	.16**
D6	Beliefs about consequences							.50**	.34**	.32**	.13*	.29**	.39**	.27**	.45**	.50**	-.30**	.52**	.42**
D7	Intentions								.35**	.22**	.15	.24**	.37**	.18**	.42**	.36**	-.31**	.46**	.47**
D8	Goals									.18**	.06	.18**	.25**	.08	.27**	.25**	-.26**	.29**	.29
D9	Innovation										.29**	.28**	.20**	.26**	.34**	.35**	-.29**	.40**	.43**
D10	Socio-political context											.17**	.19**	.36**	.24**	.15*	-.02	.15*	.12*
D11	Organization												.19**	.16**	.31**	.28**	-.27**	.19**	.20**
D12	Patient													.17**	.42**	.34**	-.22**	.33**	.36**
D13	Innovation strategy														.25**	.16**	-.02	.11	.08
D14	Social influences															.37**	-.21**	.46**	.45**
D15	Positive emotions																-.52**	.48**	.38
D16	Negative emotions																	-.37**	-.33**
D17	Behavioral regulation																		.52**
D18	Nature of the behaviors																		

Note. *, $p < .05$; **, $p < .01$
