# Supplementary material for: Measuring determinants of implementation behavior: psychometric properties of a questionnaire based on the theoretical domains framework
Source: Implement Sci. 2014 Mar 19;9:33. doi: 10.1186/1748-5908-9-33 (PMC4000005; doi:10.1186/1748-5908-9-33)
Supplement: Additional file 3 — Attenuation-corrected correlations. [file 1748-5908-9-33-S3.pdf]

Additional file 3 – Attenuation-corrected correlations

		D1	D2	D3	D4	D5	D6	D7	D8	D9	D10	D11	D12	D13	D14	D15	D16	D17	D18
D1	Knowledge		.80	.74	.40	.11	.43	.26	.19	.26	.05	.16	.17	.07	.17	.38	-.34	.33	.28
D2	Skills			.86	.47	.15	.53	.41	.21	.21	.02	.16	.26	.04	.28	.37	-.36	.47	.45
D3	Social/professional R&I				.39	.10	.53	.46	.23	.19	.10	.11	.26	.12	.31	.29	-.29	.38	.39
D4	Beliefs about capabilities					.37	.62	.46	.30	.70	.15	.38	.37	.26	.49	.55	-.42	.79	.66
D5	Optimism						.30	.10	.08	.29	.11	.28	.23	.02	.19	.37	-.18	.27	.20
D6	Beliefs about consequences							.58	.40	.42	.16	.34	.50	.32	.53	.59	-.36	.64	.51
D7	Intentions								.39	.28	.19	.28	.45	.21	.47	.41	-.35	.55	.53
D8	Goals									.23	.08	.20	.31	.09	.31	.29	-.30	.34	.34
D9	Innovation										.41	.36	.28	.35	.45	.45	-.38	.55	.57
D10	Socio-political context											.21	.26	.46	.30	.19	-.02	.20	.16
D11	Organization												.23	.19	.36	.33	-.31	.23	.24
D12	Patient													.22	.53	.43	-.28	.44	.46
D13	Innovation strategy														.30	.19	-.03	.14	.10
D14	Social influences															.43	-.25	.56	.52
D15	Positive emotions																-.61	.59	.45
D16	Negative emotions																	-.46	-.38
D17	Behavioral regulation																		.63
D18	Nature of the behaviors																		
